# A Liquid Thickener Presentation Format for the Therapeutic Management of Dysphagia—A Promising Step Forward in Addressing the Challenges Associated With Thickened Fluids in Swallowing Disorders?

**DOI:** 10.1111/1460-6984.70173

**Published:** 2025-12-25

**Authors:** Natascha Ullrich, Brenda Mossel, Sue Pownall, Jo Burke, Helena Perry, Heather Robinson, John Stephenson

**Affiliations:** ^1^ Sheffield Teaching Hospitals NHS Foundation Trust Sheffield UK; ^2^ Trisco Foods Brisbane Australia; ^3^ York and Scarborough Teaching Hospitals NHS Foundation Trust York UK; ^4^ School of Human and Health Sciences University of Huddersfield Huddersfield UK

**Keywords:** oropharyngeal dysphagia, palatability, thickened beverages, tolerance, viscosity inhibited, xanthan gum

## Abstract

**Introduction:**

Thickening fluids in routine care of patients with Oropharyngeal dysphagia (OD) can improve swallow safety but may be counteracted by reduced palatability, enjoyment, embarrassment, and isolation related to drinking thickened fluids (TFs). TFs are also associated with increased caregiver burden and significant lifestyle alterations. Thus, there persists a need to overcome these disadvantages. Precise Thick∼N INSTANT (PTI) is an innovative thickener product in viscosity‐inhibited liquid form that presents a promising step forward in addressing the challenges. This prospective multi‐centre single‐arm feasibility study of acceptability (derived from measured variables gastrointestinal (GI) tolerance, palatability, compliance and user experience) compared PTI to usual mode of care (powder) in a cohort of patients with OD.

**Methods:**

Oral fluids were thickened with PTI, adhering to standardised requirements, and were tested for palatability (primary outcome), ease of use, GI symptoms and compliance by medically diagnosed patients with OD for 14 days, following 7days under usual mode of care and a 5‐day washout period. Data was analysed descriptively, presenting effect sizes with associated precision and indicative significance testing of key variables.

**Results:**

Twenty‐four participants provided usable data. Mean overall palatability ratings revealed significantly higher palatability perceptions (*p *< 0.001) in uncorrected paired‐samples *t*‐testing for PTI‐TFs (difference in means 3.83 [95% CI 2.60 to 5.05]) on 10‐point visual analogue scale, favouring beverages thickened with PTI over usual mode of care. Compared with usual mode of care, PTI thickener showed substantive improvements in all individual palatability and satisfaction/ease of use attributes, equivalent or improved symptoms of GI and excellent levels of GI tolerance and compliance. GI side effects (e.g., nausea, bloating) were mild and of short duration.

**Conclusion:**

PTI‐TFs were more palatable, acceptable, and well tolerated in patients with OD and strongly preferred over powder thickened TFs, with improved compliance and reduced wastage. PTI is a palatable, acceptable and well‐tolerated way to support optimal hydration in adults with OD.

**WHAT THIS PAPER ADDS:**

*What is already known on this subject*

- Thickening fluids in routine care of patients with OD can improve swallow safety but can be counteracted by reduced palatability and enjoyment, embarrassment, and isolation related to drinking thickened fluids (TFs). TFs are also associated with increased caregiver burden and significant lifestyle alterations. OD concomitant with aphasia is common in some OD subpopulations; however, these patients are typically excluded from research due to the lack of aphasia friendly survey tools. There persists a need to overcome these disadvantages.

*What this paper adds to existing knowledge*

- The current investigation is the first study to directly compare acceptability (derived from the measured variables palatability, tolerance, ease of use and preference) for TFs thickened using different thickener presentation formats in the same patient cohort. The liquid thickener intervention is a palatable, acceptable, and well‐tolerated way to support optimal hydration in adults with OD. In addition, aphasia‐accessible study materials have been developed to facilitate future recruitment and data collection from this underserved subgroup.

*What are the potential or actual clinical implications of this work?*

- PTI thickened beverages are recommended for consumption by patients suffering from OD as an alternative to powdered thickened TFs. PTI is a palatable, acceptable, and well‐tolerated way to support optimal hydration in adults with OD prescribed TFs. This study also highlights an important methodological advancement in OD research related to inclusion of participants with language impairment.

## 1 Introduction

Oropharyngeal dysphagia (OD), difficult and/or disordered swallowing, is a common complication associated with stroke (Balcerak et al. 2022; Bolivar‐Prados et al. 2019) and other neurological conditions (Cheng et al. 2022), and ageing (Baijens et al. 2016; Rofes et al. 2011). The pathophysiology of OD includes mechanical deficits in the swallow response (mainly delayed laryngeal vestibule closure time and weak tongue thrust), reduced pharyngeal sensitivity, and sensory/motor central nervous impairments (Ortega et al. 2017). Traditionally, OD management is categorised into two major groups: (a) compensatory (e.g., modifications of bolus size or texture or feeding posture to improve bolus flow) or (b) rehabilitative (e.g., behavioural exercises). New strategies, including rehabilitative exercises, biofeedback, pharmacological, neuromodulation, and soft robotics, are emerging; however, further evidence of their therapeutic potential is needed (Balcerak et al. 2022; Cheng et al. 2022). Despite the emergence of new OD management strategies and recent debate challenging the clinical efficacy of texture modification (King et al. 2024; Steele et al. 2021), practice patterns suggest fluid modification to preserve oral feeding and avoid unsafe swallowing remains a primary management strategy (Werden Abrams et al. 2023; Gosa et al. 2020). Thickened fluids (TFs) increase the viscosity of the ingested fluid, thus increasing the bolus’ resistance to flow. This has advantages as a reduced flow rate allows time for air passages to close completely, and OD patients are less likely to aspirate or to experience negative aspiration‐related health consequences (Newman et al. 2016). TFs also allow improved oral motor control where a person exhibits posterior spillage due to lingual incoordination (Cichero 2019). Texture modification may be appropriate for certain patient populations where alternate strategies are contraindicated (e.g. free water protocols) or where intact motor and cognitive skills are required and need to be facilitated by care providers. Thus, TFs can be an important intervention where OD is comorbid with neurological conditions affecting cognition (e.g. stroke, Parkinson's disease, and dementia (including Alzheimer's disease)) or where TFs may facilitate safe swallowing during recovery and swallow rehabilitation (e.g., post‐acute stroke where patients aspirate a thin fluid but have a positive prognosis for swallow function rehabilitation). However, improving swallow safety with TFs may be counteracted by reduced palatability (Gallegos et al. 2021; Matta et al. 2006).

Anecdotally, patient noncompliance (e.g., patient‐initiated intake restrictions, consistency modification and premature treatment termination) is common (Balcerak et al. 2022), with caregiver dissatisfaction a reported contributing factor (Colodny 2005). Adults with OD prescribed TFs may experience disinterest, reduced enjoyment (thickening agents may negatively affect mouthfeel and flavour release) (Cichero and Lam 2014), embarrassment, and/or isolation related to drinking TFs (Wu et al. 2021). TFs may also be associated with increased caregiver burden and require significant lifestyle alterations for both patients and carer(s) (Ortega et al. 2017), affecting quality of life (QoL). Unsurprisingly, potential hazards and disadvantages associated with this management strategy include poor hydration, poor nutrition and poor QoL for both individuals with OD and their carer(s) (Steele et al. 2021; Wu et al. 2021).

In clinical practice, modification of oral fluid viscosity is typically achieved by adding powdered thickening gums (Baijens et al. 2016), which poses inherent problems (Hadde et al. 2021). The hydration properties of the thickening gums play a crucial role in the final modified texture. For example, xanthan gum (XG), a commonly prescribed powdered thickener, has the disadvantage that it easily forms agglomerates (lumps) on hydration, and high shear mixing is required to rapidly break down these agglomerates. Unsurprisingly, lumpy, aerated, aesthetically unappealing beverages are a frequently cited problem in clinical practice, leading to poor compliance with TFs (Hadde et al. 2021). Moreover, extended mixing and/or hold times are required to completely hydrate the thickener to achieve the desired consistency, and once viscosity increase has started, agitation of the fluid and therefore powder dispersion becomes increasingly more difficult, thereby exacerbating hydration problems, particularly at thicker consistencies (e.g., International Dysphagia Diet Standardisation Initiative [IDDSI] Level 3 and 4). Hydration of powdered thickeners in some applications produces undesirable interactions, limiting their use and therefore the breadth and choice of beverages available to OD patients. In the example of carbonated beverages, a significant amount of foam is produced as the powdered thickener is added and stirred (necessarily) vigorously. Powder particles provide surfaces where carbon dioxide molecules collect and form large gas bubbles that rise to the liquid surface. Consequently, the gum hydrates in the head of foam rather than the liquid (Hadde 2017), producing aesthetically unappealing beverages of mixed consistency unsuitable for individuals with OD. There persists a need for novel thickening products that overcome these disadvantages, are easy to use and yield TFs with sensory properties more comparable to non‐thickened beverages if patient satisfaction and compliance with modified texture diets is to be improved.

Precise Thick∼N Instant (PTI) is a viscosity‐inhibited liquid thickener concentrate (i.e. maintains a relatively low viscosity in situ, and when diluted on addition to a fluid, the viscosity of the target fluid is increased significantly). Having a liquid presentation format, the thickening gums in PTI are already partially hydrated, and thus, the hydration process completes rapidly after gentle agitation upon addition to a beverage. Unlike powdered counterparts, PTI dissolves fully and rapidly in a wide range of beverages (including alcoholic and carbonated) without agglomerating or the need for excessive shear. This results in aesthetically appealing TFs where the desired consistency is expressed in a short time (<30 s) without lumping or aerating. Having a liquid presentation, PTI is dosed directly into fluids by way of a precision pump, making it easier to use. Moreover, PTI is formulated to contain thickener combinations. The role of thickener combinations in manipulating hydration and altering textural and sensory properties of foods and beverages is well known (Saha and Bhattacharya 2010). Due to synergistic interactions between different thickening agents, which can alter rheological properties of the food matrix (Saha and Bhattacharya 2010), thickener combinations can create TFs with unique mouthfeel properties that differ from those of single thickeners. For example, mucoadhesion (sticky after‐feel) reported with TFs thickened with XG (Cichero and Lam 2014) may be reduced. Unpleasant mouthfeel, ‘off flavours’ (undesirable/unexpected taste) and flavour suppression associated with TF non‐compliance may be similarly altered. Thus, PTI, which contains combinations of thickening gums, may have clinical advantage in producing TFs with improved acceptability, textures and flavours closer to un‐thickened beverages. Importantly, as reported by Hadde et al. (2017), the apparent viscosities of PTI TFs are within published ranges (150‐1400 mPa.s at 50 s‐1) (Bolivar‐Prados et al. 2019), demonstrated to improve swallow safety and exhibit similar shear thinning behaviour to powdered XG TFs over the shear rates associated with oropharyngeal swallow (50–300 s^−1^) (https://swallowgateway.com/products/).

To the best of the authors’ knowledge, the current investigation is novel as it is the first to directly compare acceptability, derived from the measured variables palatability, tolerance, ease of use and preference) for TFs thickened using different presentation formats (powdered and liquid) in the same cohort of patients presenting with OD. Moreover, thickeners are commonly prescribed for patients with swallowing difficulties following a stroke. Stroke patients commonly have aphasia and thus tend to be excluded during recruitment phases of these types of studies due to a lack of aphasia‐friendly survey tools. In this study aphasia accessible study information was developed with the assistance of the hospital Patient and Public Involvement (PPI) committee to support recruitment of participants who presented with aphasia.

This study aimed to directly compare palatability, ease of use and gastrointestinal (GI) tolerance of oral fluids thickened with PTI and powdered XG‐based thickeners in a cohort of patients with OD, including those with aphasia. This research further aimed to ascertain if options such as liquid thickeners have advantages in improving adherence to and compliance with TFs and thus can assist in overcoming adverse clinical outcomes commonly associated with TF diets.

## 2 Materials and Methods

*Recruitment, Screening and Eligibility Criteria*

## 3 Study Design and Participant Recruitment

This study was conducted at two NHS hospitals and community trusts in Yorkshire, UK, following the UK Policy Framework for Health and Social Care Research.

### 3.1 Recruitment Process

Participants were recruited over 5 months by the investigating Speech and Language Therapist (SLT) or a member of the wider SLT team from acute stroke wards, inpatient rehabilitation centres, and community stroke services. Eligible participants were identified through screening based on predefined criteria (Table 1) and were recruited if they provided written consent. Additionally, patients with OD related to other neurological conditions known to domiciliary SLT teams were approached for screening and potential participation. Participants were either clinically diagnosed with OD and/or diagnosed via VFFS instrumental assessment, according to the respective NHS trust protocols and were deemed by the diagnosing clinician to benefit from a modified oral diet (i.e., thickened fluids). Ineligible individuals or those who declined participation continued to receive standard care.

**TABLE 1:** Participant inclusion and exclusion criteria.

Inclusion criteria	Exclusion criteria
Adult participants 18+ years.	Participants not requiring TFs as prescribed by a qualified health practitioner after an OD diagnosis for acute or chronic swallowing difficulties.
Participants already taking adequate amounts of TFs as determined by the treating SLT. All oral fluids thickened. No participants on free water protocols	Participants requiring enteral tube feeding or parenteral nutrition, including patients requiring top‐up enteral feeding alongside oral intake
Participants using a XG‐based powdered thickener.	Participants with medical or dietary contraindication to any of the ingredients of PTI.
Participants expected to require TFs for at least 4 + weeks from the point of recruitment.	Participants currently using starch‐based thickeners which do not contain fibre (due to gastrointestinal difficulties caused by the high (>85%) fibre content of XG).
Participants who have been taking XG thickener at a prescribed IDDSI level with no reported GI difficulties.	Participants with inflammatory bowel disease or previous bowel resection (due to gastrointestinal difficulties caused by the high (>85%) fibre content of XG).
Participants able to give written consent, or witnessed verbal consent if unable to write.	Participants enrolled in any other studies concomitantly or within a month prior to entry into this study.
Participants with mild acquired communication difficulties who understand 3+ information‐carrying words as determined by the Consent Support Tool (CST).	Participants for whom the investigator has concerns regarding the ability or willingness of the patient and/or care provider to comply with protocol requirements.
Participants with the cognitive ability to engage meaningfully in conversation and who can access and retain the study information and consent.	Participants currently using a pre‐thickened beverage or previous experience of PTI.
	Participants with significant cognitive impairment.
	Participants with an acquired communication difficulty presenting with significant visual and /or hearing difficulties and having less than a 3‐keyword understanding as determined by the Consent Support Tool (CST) (Jayes and Palmer 2014).
	Participants receiving ongoing/concomitant rehabilitative therapy to address their dysphagia

### 3.2 Considerations for Aphasia Patients

Accessible study materials to support stroke patients with aphasia were developed with the Patient and Public Involvement (PPI) panel at the lead NHS Trust site. However, no aphasic patients met the inclusion criteria (read at least three key words in a sentence using the Consent Support Tool (CST) (Jayes and Palmer 2014) and none were enrolled.

### 3.3 Ethical Considerations, Clinical Oversight and Data Management

Participants continued to receive their usual clinical care throughout the study. Where patients were undergoing adjunct rehabilitation therapy, this was maintained in full. The only variable modified was the type of thickener used (standard powder vs. PTI liquid). No participant experienced a withholding of standard care.

Participant safety was monitored under the direct oversight of the Chief Investigator and Chief Researcher, both clinical speech and language therapists. Adverse event risks and reporting procedures were defined in the ethics‐approved protocol, with any adverse events documented by an appropriate qualified health professional (e.g., the participant's GP for remote participants or a nurse in non‐domiciliary settings). Participants were contacted by a clinical member of the research team every 2 days by telephone to support diary completion and to check for issues or concerns. In‐person visits were conducted at baseline (Day 1), Day 7, during the washout, during the intervention, and at Day 26. Adverse events were additionally monitored through the governance processes of both organisations involved in the multicentre trial.

Screening was conducted by SLTs within direct care teams, with no external involvement. Screening logs documented all potential participants, recruitment status, and reasons for non‐recruitment, these are summarised in the CONSORT diagram (Falci and Marques 2015).

#### 3.3.1 Consent

Participation was voluntary, and participants could withdraw from the study at any time. Participants without aphasia were provided with a standard participant information sheet and consent form. Provision for patients with aphasia was made by the investigating SLTs, instructed to follow the approach to the informed consent process as described by Palmer et al. (2013). Consent was reconfirmed at each study visit, as recommended by Good Clinical Practice Guidelines (US Food & Drug Administration 2018).

#### 3.3.2 Sample Size

**FIGURE 1:**
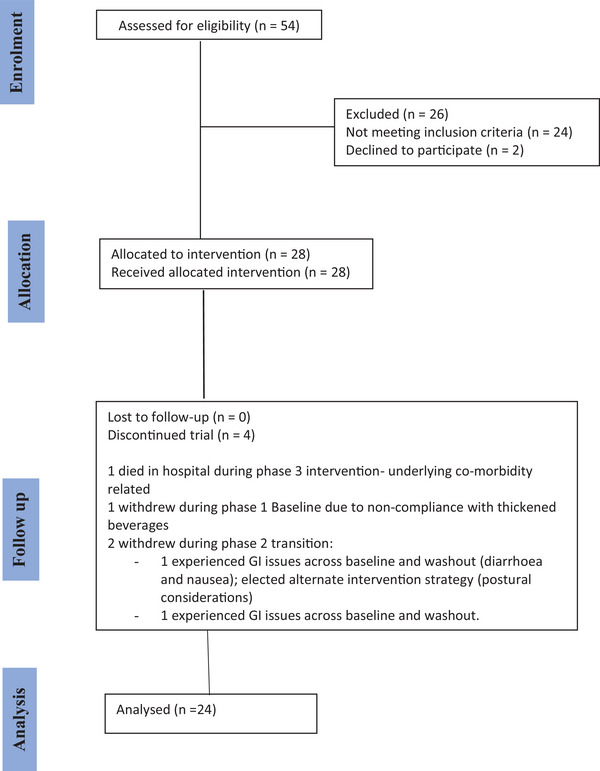
CONSORT flow diagram.

The study collected data from participants from two NHS sites, providing information on both their existing product and the new product. Participants who were unable to complete the minimum 14 days on PTI were not included in the final data analysis. Reason(s) for not completing the study are reported in the CONSORT diagram (Figure 1).

#### 3.3.3 Statistical Design

Analysis focused primarily on descriptive presentation of effect sizes (e.g., differences in outcomes associated with the PTI and usual modes of care) and associated 95% confidence intervals. As a feasibility study, the study was not powered to assess treatment effectiveness; however, preliminary uncorrected indicative significance testing was also conducted where appropriate to yield preliminary indications of effectiveness.

#### 3.3.4 Study Design

The study was a prospective multi‐centre single‐cohort, crossover design in which participants initially received standard care and were then transitioned to the intervention, where each participant acted as their own control, enabling direct comparison of outcomes between care modes. All participants remained on their clinically prescribed IDDSI thickness throughout the study, ensuring that within‐person comparisons between usual care and the intervention were not influenced by changes in fluid thickness.

The following phases were completed by all participants:

- **Phase 1. Baseline**: Post‐enrolment into the study, participants remained on their current mode of care (powdered thickener) for 7 days. During this time, a relevant medical history was taken, and current medications were recorded. Palatability, ease of use, GI tolerance and compliance were recorded over this first analysis period.
- **Phase 2. Transition**: Enrolled participants switched from their usual mode of care to thickening all their beverages with PTI over a 5‐day wash‐out period to clear the GI tract and ensure any GI issues in the study period were related to PTI. During this time, the subject and/or their carer(s) continued to record GI tolerance and compliance data in the study diaries. Data from Phase 2 were recorded to note any unexpected difficulty transitioning from one thickener to the other, but were excluded from the final analysis.
- **Phase 3. PTI intervention**: For 14 days, enrolled participants remaining in the study and/or their carer thickened all their liquids with PTI; with palatability, ease of use, GI tolerance and compliance recorded over this second analysis period
- **Phase 4**. End of study, return to baseline: After completion of the study protocol, participants returned to their usual mode of care. Participants were given the option of returning to powdered XG‐based thickener, or they could remain on PTI if the participant desired to do so.

In this study, the length of observation between phases: 7 days for baseline (usual care) and 14 days for the intervention, are unequal. This design choice reflects the fact that patients were already stable and familiar with their usual thickening regimen, so a shorter baseline was sufficient to characterise their experience. In contrast, a longer period was deliberately allocated to the intervention to allow participants time to adapt and to ensure a robust assessment of palatability, tolerance, and compliance outcomes.

During all phases, patients were given freedom to choose what beverages were thickened. Patients and carers were verbally instructed to thicken all beverages to their prescribed IDDSI consistency.

#### 3.3.5 Outcomes

Palatability was the primary outcome of the study. Palatability during Days 1–7 and 13–26 was recorded on a 6‐question survey adapted from a questionnaire developed by Flint et al. (2000) and administered at Day 7 (for the usual mode of care analysis period) and Day 26 (for the PTI analysis period). The visual analogue scale (VAS), designed to measure visual appeal, smell, taste, aftertaste, and overall pleasantness of a food product, is included in Appendix 1.

All other outcomes were considered to be secondary outcomes. Ease of use data was collected at the end of Phase 1 and Phase 3 and was recorded using an end‐anchored 5‐point Likert scale questionnaire developed to assess user satisfaction with the respective thickener's performance. A modified Usefulness, Satisfaction, and Ease of Use (USE) Questionnaire was used to measure ease of use for participants and/or carers (Lund 2001). The USE questionnaire is a valid and reliable instrument, is non‐proprietary and technology‐agnostic, and therefore can be applied to various scenarios of usability assessment (Gao et al. 2018). Additional bespoke questions to capture wastage and timeliness of beverage production (Appendix 2) were added to the survey instrument. The survey comprised 7 items measuring user satisfaction, 1 item measuring wastage and 2 items measuring preparation time.

Gastrointestinal (GI) tolerance data were recorded daily and collected using daily questionnaires, using a modified version of a 17‐item questionnaire by Bovenschen et al. (2006). Six symptoms featured in the original questionnaire were repurposed for the current investigation: flatulence, abdominal rumbling, bloating, abdominal pain, nausea, and vomiting. These symptoms were selected based on those assessed by Carabin et al. (2009), who evaluated the effects of thickening gum fibres on GI tolerance. The descriptors used to assess the intensity and frequency of symptoms were *none*, *mild*, *moderate*, *quite a lot*, *severe*, *very severe* and *unbearable*; scored from 0 to 6 points respectively. Further questions regarding stool frequency and consistency, adapted from the Bristol Stool Scale (BSS) (Lewis and Heaton 1997), were also assessed. The full set of items is reported in Appendix 3 and Appendix 4 (Aphasia friendly [AF]).

## 4 Compliance

Compliance data was collected using a nutritional intake chart widely used in health settings. Participants and/or carer(s) were asked to record quantities of drinks prepared and drinks wasted daily. In addition, average daily quantities of TFs consumed on usual mode of care and during PTI intervention were compared to the volume of TFs required to meet 100% of the European Food Safety Authority (EFSA) water adequate intake (AI), excluding water from food (EFSA Panel on Dietetic Products, Nutrition, and Allergies (NDA) 2010); defined as 2.0 L/day for adult females and 2.5 L/day for adult males, assuming that the contribution of food to total dietary water intake is 20%–30% (EFSA Panel on Dietetic Products, Nutrition, and Allergies (NDA) 2010). Compliance calculations used in this study calculated fluid intake based on 20% of water being consumed in food.

## 5 Results

### 5.1 Participant Characteristics

The CONSORT flow diagram of patients eligible, recruited, numbers followed up and included in analysis is shown in Figure 1. Twenty‐eight volunteers with OD were recruited into the trial, comprising 20 patients from the lead site and 8 from the second site. Three patients from the lead site and 1 patient from the other site were subsequently withdrawn from the trial and did not provide usable data for either period of analysis. Analysis was conducted on the remaining 24 patients (referred to as ‘completing patients#x02019;).

The mean age of completing patients was 75.8 years (SD 9.49 years; range 53 to 88 years). Twenty‐one patients (87.5%) were aged 65 years or more. Thirteen patients (54.2%) were male, and 11 patients (45.8%) were female. The usual mode of care product was *Nutricia Nutilis* for the lead site patients and *Nestle Resource Thicken Up* for the second site patients. Demographic and clinical features of participants at enrolment are shown in Table 2.

**TABLE 2:** Participant demographic and clinical features of patients with dysphagia with a prescribed powdered XG thickener at enrolment.

Location	ID	Gender	Age (years)	Associated medical aetiology	Clinical care setting
Lead site	SH001	Male	73	Repeat fluid penetration and Aspiration	Domiciliary
Lead site	SH002	Female	86	CVA	Domiciliary
Lead site	SH003	Male	81	CVA	Domiciliary
Lead site	SH004^a^	Male	75	Parkinson's disease	Domiciliary
Lead site	SH005	Female	72	Parkinson's disease	Domiciliary
Lead site	SH006	Female	72	Dysphagia secondary to COPD	Domiciliary
Lead site	SH007^a^	Male	82	CVA	Domiciliary
Lead site	SH008	Male	77	COPD	Domiciliary
Lead site	SH009	Male	88	CVA	SPARC
Lead site	SH010	Male	84	Repeat fluid penetration and Aspiration	Domiciliary
Lead site	SH011^a^	Female	73	CVA, COPD, GORD	Care home facility
Lead site	SH012	Female	60	CVA	Domiciliary
Lead site	SH013	Female	87	CVA	Acute stroke hospital setting
Lead site	SH014	Female	65	Multiple Sclerosis	Domiciliary
Lead site	SH015	Male	71	H&N Cancer	Domiciliary
Lead site	SH016	Male	53	Idiopathic bilateral vocal cord palsy, acute haemorrhagic gastritis	Domiciliary
Lead site	SH017	Male	84	H&N Cancer	Domiciliary
Lead site	SH018	Male	85	Parkinson's Disease	Domiciliary
Lead site	SH019	Female	76	CVA	Domiciliary
Lead site	SH020	Male	79	Thyroidectomy, submandibular tumour, Bowen's disease, hiatus hernia	Domiciliary
Secondary site	YK001	Female	75	MND	Domiciliary
Secondary site	YK002	Female	55	Multiple Sclerosis	Domiciliary
Secondary site	YK003	Male	80	MND	Domiciliary
Secondary site	YK004	Female	77	Multiple System Atrophy	Domiciliary
Secondary site	YK005	Female	77	MND	Domiciliary
Secondary site	YK006^a^	Male	86	Oesophagitis	Domiciliary
Secondary site	YK007	Male	75	MND	Domiciliary
Secondary site	YK008	Female	87	H&N Cancer	Domiciliary

Abbreviations: COPD, chronic obstructive pulmonary disease; CVA, cerebrovascular accident; GORD, gastro‐oesophageal reflux disease; H&N Cancer, head and neck cancer; MND, motor neurone disease.

^a^Patient withdrew from the study.

#### 5.1.1 Palatability

**FIGURE 2:**
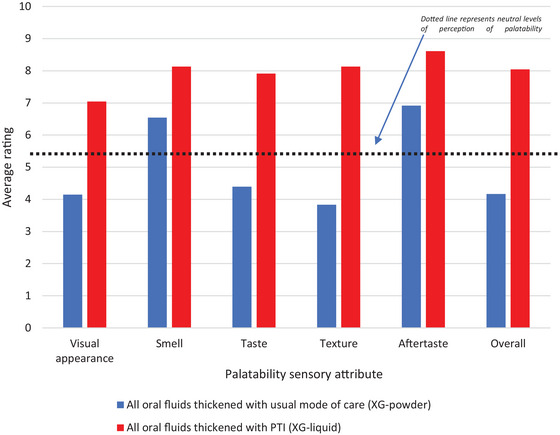
Summary of palatability attribute ratings: Usual mode of care and PTI intervention.

Full palatability data under PTI and usual modes of care were collected from 23 completing patients (95.8%). One patient provided data under usual mode of care only. Fluids thickened with PTI scored consistently higher on average than fluids thickened with usual mode of care for all measured sensory attributes (visual appearance, smell, taste, texture, aftertaste, and overall pleasantness). Oral fluids thickened with PTI were found to be acceptable with mean palatability ratings of 6.95 or above in all domains; with mean overall pleasantness rating of 8.37 (SD 1.49); compared to 4.00 (SD 2.34) for fluids thickened under usual mode of care. Paired samples t‐tests conducted on patients providing palatability under both modes of care revealed that ratings of palatability characteristics for oral fluids thickened with PTI were, in all cases, significantly higher than corresponding ratings given to oral fluids thickened under usual mode of care. Pre‐post differences in mean scores were substantive (2 points or more) for all palatability attributes, and particularly so for the attributes of taste, texture and overall perception; all of which recorded pre‐post improvements of over 4 points on the 10‐point VAS scale (Table 3; Figure 2).

**TABLE 3:** Palatability by sensory attribute over trial duration.

	Mean Palatability Rating by sensory attribute (mean (SD))					
Product	Visual appearance	Smell	Taste	Texture	Aftertaste	Overall pleasantness
Usual mode of care (*n* = 24)	4.15 (SD 2.43)	6.54 (SD 2.12)	4.40 (SD 2.56)	3.83 (SD 2.75)	6.92 (SD 2.89)	4.17 (SD 2.78)
PTI (*n* = 23)	7.04 (SD 2.53)	8.13 (SD 1.98)	7.91 (SD 1.86)	8.13 (SD 2.13)	8.61 (SD 2.34)	8.04 (SD 2.20)
Difference in means^1^means^a^						
Difference in means^a^ (95% CI)	2.72 (1.41, 4.03)	1.70 (0.51, 2.89)	3.54 (2.45, 4.64)	4.35 (2.92, 5.77)	1.83 (0.77, 2.88)	3.83 (2.60, 5.05)
*p* Value	<0.001	0.007	<0.001	<0.001	0.002	<0.001

^a^ Expressed in terms of (rating under PTI—rating under usual mode of care).

#### 5.1.2 Ease of Use

Valid responses were received from all completing patients to each of the user satisfaction items and the single wastage item, with negligible levels of missing data for the preparation time items. For all the user satisfaction items, higher scores represented more positive perceptions of ease of use, except Item 2 (‘*I found using the thickener unnecessarily complex’*), which was reverse‐coded; hence, lower scores represented more positive perceptions of ease of use for this item. For the wastage items, lower scores represented lower levels of wastage and hence more satisfactory outcomes. For the preparation time items, lower scores represented lower preparation times, and hence more satisfactory outcomes. Table 4 summarises the median and IQR item scores under both modes of care, revealing substantive improvements on the ease of use, wastage and preparation items under PTI compared with usual mode of care.

**TABLE 4:** Summary of responses to ease of use items.

Item	Usual mode of care (median; IQR)	PTI (median, range)
Ease of use/satisfaction 1	3 (2–4)	5 (5–5)
Ease of use/satisfaction 2	2.5 (2–4)	1 (1–2)
Ease of use/satisfaction 3	2.5 (2–4)	5 (5–5)
Ease of use/satisfaction 4	4 (4–4)	5 (5–5)
Ease of use/satisfaction 5	4 (3–4)	5 (5–5)
Wastage 1	2 (2–3)	1 (1–1)
Preparation 1	2 (1–3)	1 (1–1.5)
Preparation 2	2 (1–3)	1 (1–1)

#### 5.1.3 Gastrointestinal (GI) Tolerance

A total of 2761 valid GI symptom evaluations were obtained out of a potential 3024 evaluations over the entire study period, excluding the washout period (8.7% missing data).

PTI was well tolerated with mild GI symptoms in a minority of participants compared to usual mode of care. Mean number of daily symptoms, mean symptom severity (using the modified tool adapted from Bovenschen et al. (2006) and number of patients reporting symptoms were lower under PTI than in usual mode of care for all measured symptoms, despite the longer duration of the PTI mode of care.

Flatulence and abdominal rumbling were the most prevalent symptoms in both phases of the analysis. No patients experienced vomiting in either phase of the study. Reported symptoms were, on average, mild in nature and generally ranged from ‘mild’ to ‘moderate’ in severity in both the PTI and usual modes of care, with isolated instances of symptoms reported to be ‘quite a lot’ and ‘very severe’ (Table 5).

**TABLE 5:** Summary of GI symptoms.

		Symptom					
Treatment	Outcome	Bloating	Abdominal rumbling	Flatulence	Abdominal pain	Nausea	Vomiting
Usual mode of care^1^	Number (%) of patients reporting symptom	7 (29.2%)	10 (41.7%)	11 (45.8%)	5 (20.8%)	2 (8.3%)	0 (0.0%)
	Total number of episodes	19	20	56	14	7	0
	Mean episodes per patient per day^3^	0.118	0.125	0.359	0.0870	0.0438	0.00
	Mean severity (SD)	0.20 (0.51)	0.16 (0.35)	0.55 (0.83)	0.13 (0.31)	0.08 (0.37)	0.00 (0.0)
PTI treatment	Number (%) of patients reporting symptom	5 (20.8%)	8 (33.3%)	10 (41.6%)	5 (20.8%)	1 (4.2%)	0 (0.0%)
	Total number of episodes	19	21	77	12	1	0
	Mean episodes per patient per day	0.0633	0.0698	0.263	0.0399	0.00333	0.00
	Mean severity (SD)	0.15 (0.57)	0.076 (0.14)	0.55 (0.96)	0.084 (0.21)	0.003 (0.002)	0.00 (0.0)

The effect of treatment on severity of symptoms was low for bloating (Cohen's *d* = 0.09) and flatulence (Cohen's *d* = 0.006); and moderate for other symptoms (Cohen's *d* = 0.30 for abdominal rumbling; Cohen's *d* = 0.35 for abdominal pain; Cohen's *d* = 0.40 for nausea).

#### 5.1.4 Stool Frequency and Consistency

Valid stool frequency figures were obtained from 439 out of 457 potential patient‐days (3.9% missing data). The baseline daily number of stools was largely unchanged during the intervention (Table 6). Abnormal stool consistency (points 1, 2, 5, 6 or 7 on the Bristol Stool Scale) was reported on 46 out of 113 reported patient‐days (40.7%) with 1 or more stools reported under usual mode of care, and in 85 out of 233 reported patient‐days (36.4%) with 1 or more stools reported under PTI. Diarrhoea (points 5, 6 or 7 on the Bristol Stool Scale) was reported on 27 out of 113 reported patient‐days (23.9%) under usual mode of care and in 59 out of 233 reported patient‐days (25.3%) under PTI. Uncorrected *Z*‐tests for the equality of two proportions revealed no evidence that proportions of stool abnormalities or diarrhoea under the two modes of care were different (*Z* = 0.760, *p* = 0.447 for abnormal stool consistency; *Z* = 0.288, *p* = 0.773 for diarrhoea).

**TABLE 6:** Summary of Stool frequency and consistency.

Treatment	Stool frequency (mean (SD)	Abnormal stool consistency^a^ incidences per patient‐days	Abnormal stool consistency (diarrhoea)^b^ incidences per patient‐days
Usual mode of care	1.16 (0.64)	46/113 (40.7%)	27/113 (23.9%)
PTI treatment	1.30 (0.68)	85/233 (36.4%)	59/233 (25.3%)

^a^ Bristol stool scale points 1, 2, 5, 6, 7.

^b^ Bristol stool scale points 5, 6, 7.

#### 5.1.5 Compliance

The cohort average daily thickened fluid intake for beverages thickened with PTI was greater at 1.35L (SD 0.41L) than for usual mode of care at 1.21L (SD 0.45L). This effect was observed in both male and female patients, with higher percentage changes from usual mode of care in female patients. Under usual mode of care, 2 participants (1 male, 1 female; 8.3% of completing patients) met the minimum European Food Safety Authority (EFSA) water adequate intake (AI), excluding water from food. Under PTI mode of care, 4 participants (1 male, 3 females; 16.7% of completing patients) met the EFSA minimum intake. The higher compliance rates in female participants in a study with negligible difference in male and female intake reflect the higher minimum intake guidelines associated with male patients.

## 6 Discussion

This study compared the palatability, ease of use, GI tolerance, and patient compliance of TFs thickened with different presentation formats (powder and liquid) in 24 participants with varying underlying aetiologies prescribed TFs for OD management. While the role of powdered XG‐based thickeners in modifying fluid consistency for OD is well documented (Gosa et al. 2020, Falci and Marques 2015), emerging evidence suggests that liquid presentation formats are also effective (Hadde et al. 2021). To our knowledge, this is the first study to directly compare the two formats within the same patient cohort.

Our findings indicate a strong preference for the PTI thickener. Patients rated palatability significantly higher for PTI‐TFs, with a mean overall palatability score of 8.04 on a 10‐point visual analogue scale (VAS). Individual palatability attributes were also consistently rated higher compared to beverages thickened with powdered thickeners (*p* < 0.001 in preliminary uncontrolled indicative significance tests). Unpleasant mouthfeel, ‘off flavours’ (undesirable/unexpected taste) and flavour suppression were improved under the PTI intervention. Terms used by study participants to describe PTI thickened drinks included ‘silky smooth when swallowed’ and ‘no “sludge” in the bottom of the cup’ (Ullrich et al. 2024). The response to the taste of thickened drinks included perceptions that the taste of different drinks was not neutralised by the intervention. Specifically, ‘gives a much better and natural drink’. This was not unexpected as PTI is formulated to contain thickener combinations known to create TFs with unique mouthfeel properties different from those of single thickeners. Interactions between different thickening agents can alter rheological properties of the food matrix, such as lubrication (reducing sliminess) or cohesive properties (impacting ease of swallowing) (Saha and Bhattacharya 2010). Additionally, PTI demonstrated substantial improvements across all satisfaction and ease‐of‐use measures. Patient noncompliance with TFs is well recognised, with dissatisfaction primarily attributed to texture, taste and visual appeal (Cichero and Lam 2014, Cox 2021, McCurtin et al. 2018, Shim et al. 2013). Caregiver perceptions of poor palatability (Du Plessis et al. 2016) and the inconvenience of preparing TFs (McCurtin et al. 2018, Shim et al. 2013) further contribute to low adherence. Many individuals describe TFs as unpleasant, leading to reduced TF intake or noncompliance with prescribed thickness. In a qualitative study on stroke patients, McCurtain et al. (2018) reported 93% of participants expressed dissatisfaction with TFs, often using strong negative descriptors such as ‘awful’ and ‘vile’. Many also reported a suppression of flavours, leading to a dissatisfying drinking experience. Given this context, our study's findings—demonstrating higher patient‐reported palatability, improved compliance, and reduced wastage with PTI—support the conclusion that a liquid thickener can significantly enhance both patient and caregiver experiences with TFs. These results align with prior studies on PTI‐TFs. In a hospital‐based case study, Du Plessis et al. (2016) found that 94.12% of nursing staff (*n* = 16) were satisfied or extremely satisfied with PTI, and all staff reported that PTI improved patient care. Increased fluid intake among patients using liquid thickeners and improved compliance and satisfaction (Hadde et al. 2021, Viñas et al. 2022) have also been reported. Consistently, in our study, 90% of participants who completed the protocol and still required TFs opted to continue using PTI post‐study. Information of what type of fluids were thickened was outside the scope of the current research. The ability of the liquid presentation format to make thicken beverages with known sensory modulation (e.g., carbonated), however, may be a mediating variable explaining why PTI substantively improved patient experience in this study. Future research is needed to confirm this hypothesis.

In terms of fluid intake, our cohort demonstrated higher daily consumption of thickened beverages (mean 1.35L, SD 0.41L) compared to powders (mean 1.21L, SD 0.45L). This is notable given that prior studies have reported lower intake levels. For example, Hibberd (2011) observed daily consumption of 680–900 mL among nursing home residents using powdered XG thickeners. In our study, compliance with the European Food Safety Authority (EFSA) water Adequate Intake (AI), excluding water from food, was also higher with PTI. While only 8.3% of participants (2/24) met EFSA guidelines under powdered TFs, 16.7% (4/24) met these criteria under PTI. Suboptimal fluid intake among OD patients is widely reported (Cox 2021, Shim et al. 2013, Viñas et al. 2022), with dehydration rates ranging from 19% to 100% (Viñas et al. 2022). The increased fluid intake observed in our study suggests potential patient benefits associated with PTI.

In addition to improving compliance, PTI was well tolerated. Participants reported lower GI symptom severity compared to powder thickened TFs, with no significant changes in stool frequency, stool abnormality, or incidence of diarrhoea. These findings are consistent with prior studies assessing the acceptance and GI tolerance of XG‐based powdered thickeners. For instance, Hibberd (2011) observed no GI intolerance symptoms over a 2‐week period in elderly OD patients using powdered XG thickeners. Similarly, in a 14‐day study of 16 OD patients using a liquid thickener, beverages were well accepted and tolerated (Tomsen et al. 2023). Reported GI symptoms included a 33% increase in burping and flatulence and a 6% increase in abdominal pain, but no significant changes in vomiting, retching, stool frequency, or stool consistency (Tomsen et al. 2023)—similar to our study findings.

Finally, this study also highlights an important methodological advancement in dysphagia research related to participant inclusion. Language impairment frequently co‐occurs with OD following ischemic stroke; however, most OD research excludes these individuals due to the lack of AF survey tools. To address this gap, we developed AF study materials to facilitate recruitment and data collection in this sub‐population. Although no participants with co‐occurring aphasia and OD met the inclusion criteria during our recruitment window, the study materials developed may serve as useful templates for future research to better capture data from this underserved subgroup.

### 6.1 Limitations

All participants remained on their clinically prescribed IDDSI thickness throughout the study, ensuring that within‐person comparisons between usual care and the intervention were not influenced by changes in thickness. However, we did not systematically record or report the specific IDDSI levels across the cohort. As a result, we cannot describe the distribution of thickness levels or assess whether the intervention effect varied according to level. This omission does not affect the internal validity of our self‐controlled design but limits external interpretability and the ability to generalise findings across different IDDSI levels. Because this was a feasibility trial with a small sample size, recording and analysing outcomes by individual IDDSI level was not considered relevant, as the study was not powered to detect level‐specific effects. Nonetheless, this limitation highlights an opportunity for future research to power a larger study specifically designed to investigate whether intervention effects differ by IDDSI level.

As the study was conducted remotely, the potential for response bias cannot be excluded. Participants consumed their usual diet during the study, which may have contributed to some GI issues reported. In clinical practice, people are prescribed TFs for months or years and ideally consume all their daily oral hydration thickened. In this study, participants consumed TFs daily with PTI for 14 days. Results therefore, might not reflect taste fatigue that might occur with consumption over longer timeframes.

The survey questionnaires used in this study were not all fully validated instruments; in some cases adaptations of validated tools. Data collected, including compliance diary entries self/carer‐administered in the home and symptom self‐assessments, were self‐reported with limited supervision of the data collection, which may have introduced some inaccuracies. Information on the specific types of fluids thickened was not collected, which could have influenced palatability and tolerance outcomes. The ability of the liquid presentation format to thicken beverages with known sensory modulation (e.g., carbonated) may therefore be a mediating variable explaining the observed improvements in patient experience with PTI.

Finally, the study cohort was heterogeneous, reflecting real‐world practice and improving generalizability. However, the pilot nature and limited sample size precluded robust subgroup analyses or stratification by severity. This again points to the need for larger, powered studies to explore subgroup effects and further validate findings.

## 7 Conclusions

Compliance rates and palatability scores for PTI TFs were high and sustained over 14 days for patients with OD with differing underlying aetiologies. Any reported non‐compliance appeared unrelated to the specific thickener product, as it did not vary significantly across products (powder and liquid intervention). GI side‐effects were uncommon, mild, and intermittent. Improved compliance and reduced wastage under the PTI regime, higher reported scores for palatability and substantive improvements in all patient and/or caregiver satisfaction/ease metrics for the PTI product support the conclusion that the liquid thickener substantively improves both caregiver and patient experiences with TFs. Thus, the liquid thickener is a palatable, acceptable, and well‐tolerated way to support optimal hydration in adults with OD.

## Funding

This work was supported by Trisco Foods Pty Ltd. The sponsor played no role in data collection or analysis.

## Ethics Statement

Ethics approval was granted by the London—Camberwell St Giles Research Ethics Committee (REC) and the study approved by the Health Research Authority (HRA) and Health and Care Research Wales (HCRW) (REC, HRA and HCRW Reference 22/LO/0471.)

## Conflicts of Interest

Brenda Mossel is an employee of Trisco Foods. The remaining authors declare no conflicts of interest.

## Data Availability

All relevant data are summarised in the paper, and supporting information files are held in accordance with confidentiality, data collection, handling and record keeping as outlined in ethics protocol number STH22185. Data is available from the Authors on reasonable request.

## Appendix 1 Palatability Questionnaire Items

### 1.1

This questionnaire is designed to assess how much you enjoyed beverages thickened with [insert thickener].

Please make a mark on the lines under each title according to how you felt about each aspect of the beverages.

## Appendix 2 Satisfaction/Ease of Use Questionnaire Items

### 2.1

This questionnaire is designed to assess how long it took to safely thicken beverages and how satisfied you were making beverages thickened with [insert thickener]. Please make a mark in the box under each title according to how you felt about each aspect of the beverages. For wastage, please write in the corresponding box the average number of times the beverage was wasted each week. For preparation time please write in the corresponding box the actual time to prepare the beverage.

**Table:**

**User satisfaction**	**Strongly disagree**	**Disagree**	**Neither agree nor disagree**	**Agree**	**Strongly agree**
Overall, I am satisfied with how easy it is to use [insert thickener]					
I found using [insert thickener] unnecessarily complex					
I thought [insert thickener] was easy to use					
I was able to thicken beverages quickly using [insert thickener]					
I learnt to use Precise ThickN Instant very quickly					
I am very comfortable/confident using [insert thickener]					
[insert thickener] produced thickened beverages that were not smooth with lots of visible lumps that could not be easily dissolved or required considerable mixing to ‘stir away’ –					
In still non‐carbonated beverages [insert thickener] produced thickened beverages that were appetising and pleasing to look at (i.e., not aerated or with visible powder lumps)					
**Wastage**	**Never**	**Occasionally (less than 3 times a week)**	**Three times a week**	**Once a week**	**Once a day**
Overall because of poor mixing or wrong consistency when using [insert thickener] I threw a beverage | away					
**Preparation time**	**<30 s**	**30–60 s**	**1–2 min**	**2–4 min**	**>4 min**
The average mixing time to produce a beverage that wasn't lumpy, and all the thickener was dispersed using [insert thickener] was					
The average time I had to wait for the beverage to meet the right consistency (correct IDDSI level) using [insert thickener] was					

## Appendix 3 GI Items

### 3.1

This questionnaire assesses gastrointestinal symptoms. Please mark in the circle under each description the intensity or frequency of the gastrointestinal symptoms you have experienced over the last 24 h.
